# A Sco protein among the hypothetical proteins of *Bacillus lehensis* G1: Its 3D macromolecular structure and association with Cytochrome C Oxidase

**DOI:** 10.1186/1472-6807-14-11

**Published:** 2014-03-19

**Authors:** Soo Huei Tan, Yahaya M Normi, Adam Thean Chor Leow, Abu Bakar Salleh, Roghayeh Abedi Karjiban, Abdul Munir Abdul Murad, Nor Muhammad Mahadi, Mohd Basyaruddin Abdul Rahman

**Affiliations:** 1 Center for Enzyme and Microbial Biotechnology (EMTECH), Faculty of Biotechnology and Biomolecular Sciences, Universiti Putra Malaysia, Serdang, Selangor 43400, Malaysia; 2 Department of Chemistry, Faculty of Science, Universiti Putra Malaysia, Serdang, Selangor 43400, Malaysia; 3 School of Biosciences and Biotechnology, Faculty of Science and Technology, Universiti Kebangsaan Malaysia, 43600 UKM, Bangi, Selangor, Malaysia; 4 Malaysia Genome Institute, Ministry of Science, Technology and Innovation, Jalan Bangi, Kajang, Selangor 43000, Malaysia

**Keywords:** Hypothetical proteins, Bleg1_2507, Sco, Thioredoxin, Copper binding, Redox reaction, Cytochrome c oxidase

## Abstract

**Background:**

At least a quarter of any complete genome encodes for hypothetical proteins (HPs) which are largely non-similar to other known, well-characterized proteins. Predicting and solving their structures and functions is imperative to aid understanding of any given organism as a complete biological system. The present study highlights the primary effort to classify and cluster 1202 HPs of *Bacillus lehensis* G1 alkaliphile to serve as a platform to mine and select specific HP(s) to be studied further in greater detail.

**Results:**

All HPs of *B. lehensis* G1 were grouped according to their predicted functions based on the presence of functional domains in their sequences. From the metal-binding group of HPs of the cluster, an HP termed Bleg1_2507 was discovered to contain a thioredoxin (Trx) domain and highly-conserved metal-binding ligands represented by Cys69, Cys73 and His159, similar to all prokaryotic and eukaryotic Sco proteins. The built 3D structure of Bleg1_2507 showed that it shared the βαβαββ core structure of Trx-like proteins as well as three flanking β-sheets, a 3_10_ –helix at the N-terminus and a hairpin structure unique to Sco proteins. Docking simulations provided an interesting view of Bleg1_2507 in association with its putative cytochrome c oxidase subunit II (COXII) redox partner, Bleg1_2337, where the latter can be seen to hold its partner in an embrace, facilitated by hydrophobic and ionic interactions between the proteins. Although Bleg1_2507 shares relatively low sequence identity (47%) to BsSco, interestingly, the predicted metal-binding residues of Bleg1_2507 i.e. Cys-69, Cys-73 and His-159 were located at flexible active loops similar to other Sco proteins across biological taxa. This highlights structural conservation of Sco despite their various functions in prokaryotes and eukaryotes.

**Conclusions:**

We propose that HP Bleg1_2507 is a Sco protein which is able to interact with COXII, its redox partner and therefore, may possess metallochaperone and redox functions similar to other documented bacterial Sco proteins. It is hoped that this scientific effort will help to spur the search for other physiologically relevant proteins among the so-called “orphan” proteins of any given organism.

## Background

Hypothetical proteins (HPs) are generally known as proteins of unknown structures and functions [1]. They constitute approximately 25% of any sequenced genome even in simple, model organisms such as *Escherichia coli, Bacillus subtilis* and *Saccharomyces cerevisiae*[2]. Annotating their biological function has remained a challenge due to their low sequence and structural homology to other known proteins [3]. This in turn creates difficulties in attempts to understand a biological system as a whole due to the information gaps posed by these “orphan” proteins. Therefore, efforts in trying to unravel their structures and functions are crucial in filling these information gaps for any particular system and hence, should be encouraged. To deepen our understanding on these proteins, *in silico* methods of analysis are the most time and cost effective in generating a wealth of reliable and useful information on HPs ranging from their localization, properties to their possible structures and functions. It also provides a quick means to preliminary screen and chooses potential HPs for subsequent downstream *in vitro* and *in vivo* experiments, particularly to initiate structural genomic projects in the future [4].

With the wealth of information which can be generated using the *in silico* approach, this present study highlights the utilization of various *in silico* tools and methods to functionally predict and cluster all HPs present in the complete genome of a locally isolated alkaliphile, *Bacillus lehensis* G1, with the purpose of establishing a platform to mine and later select specific HPs to be studied further in greater detail. Whilst the genome of this alkaliphile has been completely sequenced [5], the structural and functional omics of this extremophile is not well characterised, particularly its HPs. Hence, our attempt at firstly predicting their functions and clustering them accordingly will serve as a platform in mining and selecting specific HPs to be studied further in greater detail. Stemming from this effort, our attention was led to a particular HP, Bleg1_2507, in the metalloprotein category of the cluster. This HP contained a Sco1 domain of the Thioredoxin (Trx) superfamily and showed up to 50% of sequence identity to other bacterial HPs and 47% to bacterial Sco proteins.

It is important to note that Sco proteins are present in both prokaryotic and eukaryotic organisms and are required for the proper assembly of cytochrome c oxidase (COX), a terminal enzyme in the respiratory chain [6]. Sco was suggested to be involved in the delivery of copper ion to COX complex [7]–[9]. To date, the only bacterial Sco1 protein which was structurally studied and analysed at length is from *Bacillus subtilis*[6] while its eukaryotic counterpart is from yeast [10], human [11] and plant [12]. Sco proteins have garnered importance in recent years due to their roles in the correct assembly of the copper center (CuA) of COX subunit II (COX II). Improper assembly of COX has been reported to cause fatal infantile encephalopathy due to mutations in *Sco1* and *Sco2* genes in humans [11,13].

Due to the physiological relevance of Sco proteins, we embarked on the task of building the structure of HP Bleg1_2507 of *B. lehensis* G1 via homology modelling to investigate the structural similarities and differences between this protein with Sco1 protein from *B. subtilis* (BsSco), yeast and human. As Sco1 was suggested to be involved in the delivery of copper ion to COX complex, specifically COXII [7]–[9], we subsequently mined the genome of *B. lehensis* G1 for the sequence encoding COXII by performing a BLASTP search. As a result, Bleg1_2337 was retrieved and its structure was built via homology modelling. Docking of both HP Bleg1_2507 and Bleg1_2337 models was performed to investigate their possible interaction. Based on the results obtained, the possible structure, function and mechanism of HP Bleg1_2507 from *B. lehensis* G1 are duly discussed. Lastly, the structure of HP Bleg1_2507 of *B. lehensis* G1 was compared to other Sco proteins to highlight its similarity and distinctness.

## Methods

### Domain and sequence similarity analysis of HPs of *B. lehensis* G1: Development of an HP cluster

All sequences encoding HPs in the genome of *B. lehensis* G1 were firstly subjected to INTERPROSCAN [14] and Conserved Domain Search (NCBI-CDD) [15] analyses to detect possible functional domains within the sequences. Sequence similarity of all the HPs with other proteins in the database was investigated also using BLASTP [16]. Based on the predicted functional domains and sequence similarity, an HP cluster was built to categorize the proteins accordingly. A scan on HPs which showed acceptable similarity to other proteins with low e-value was carried out and the potential candidate was chosen for further analyses and structure prediction.

### Sequence analysis of selected HP (Bleg1_2507) and putative Cytochrome c Oxidase subunit II, COXII (Bleg1_2337)

From the above scan, a particular HP in the metal-binding group of proteins encoded by *bleg1_2507* gene was chosen for further analyses based on the presence of the conserved copper chaperone Sco1 domain related to Trx-like superfamily. Firstly, the possible presence of consensus amino acids pattern in Bleg1_2507 sequence was identified using ScanProsite [17]. Important and metal-binding residues were subsequently identified using Consurf [18] and MetalDetector v 2.0 [19] respectively. The physicochemical aspect of the protein such as its theoretical pI value was investigated using ProtParam [20]. SigCleave from EMBOSS [21] was used to investigate the possible presence of signal peptide in Bleg1_2507, while the evolutionary relatedness of the protein with other known proteins was investigated using PHYLIP [22].

Subsequently, a genome-wide scan for Bleg1_2507 redox partner i.e. cytochrome c oxidase subunit II (COX II), was performed. As a result, Bleg1_2337, a putative COXII protein was identified from the genome sequence of *B. lehensis* G1. Further analyses similar to the ones mentioned above were performed on Bleg1_2337.

### Homology modeling of HP Bleg1_2507 and Bleg1_2337

PSI-BLAST [16] search against Protein Data Bank (PDB) was performed to retrieve potential templates for Bleg1_2507 and Bleg1_2337 model construction. Subsequently, Multiple Sequence Alignment (MSA) of the templates with the query sequences was performed using ClustalW [23] to determine the degree of similarity and conservation of specific motifs and amino acids in the sequences. 3D models of both proteins were developed via homology modeling using MODELLER 9v10 [24]. The best built models for Bleg1_2507 and Bleg1_2337 were chosen based on their lowest Discrete Optimized Protein Energy (DOPE) values and GA 341 score of one, which signify that the models resemble the native structure and hence, reliable. The generated models were visualized by PYMOL [25]. To aid assessment and ease discussion on the models, a color gradient scheme representing different levels of hydrophobicity of the amino acid side chains [26] were utilized. Further confirmation on the validity of the models was made based on their Root Mean Square Deviation (RMSD) calculated by superimposing them with their respective templates using Chimera USCF 1.6.1 [27].

### Model refinement and validation

Refinement of the built models was performed using FoldX [28] (RepairPDB) to remove Van Der Waals clashes and bad contacts of amino acid side chains. Subsequently, structural evaluation and stereochemical analyses of the refined models were performed using ERRAT which sets a 95% confidence limit being the cut-off value to evaluate any incorrect residues present in the protein structure based on the average of six atomic interactions in the protein i.e. ƒ(CC), ƒ(CN), ƒ(CO), ƒ(NN), ƒ(NO) and ƒ(OO) [29]. Further supporting analysis to evaluate the models was performed using PROCHECK Ramachandran plot [30].

### Docking of Bleg1_2507 and Bleg1_2337 models and energy minimization of protein complex

Both of the models were docked by using Cluspro v 2.0 [31] to predict protein interaction. Subsequently, the docked protein complex was refined using AMBER force in YASARA [32].

## Results and discussion

### Domain and sequence similarity analyses and clustering of HPs of *B. lehensis* G1

Based on the BleG1DB v1.0 complete genome of the locally isolated alkaliphilic *B. lehensis* G1 (released Aug 24th, 2012), the 3.5 Mbp genome consisted of 4021 predicted genes, in which 2819 of them encode proteins with putative functions while 1202 of them encode uncharacterized, hypothetical proteins [http://27.126.156.144/]. INTERPROSCAN analysis on all the HPs revealed that 51.5% of them were predicted with functions, while 48.5% were of unknown functions and hence categorized as unknown proteins (Table 1). In the category of proteins with predicted functions, majority of the HPs (286 in total) were signal peptides, in which 221 of them were transmembrane peptides. Other than these signal peptides, there were other 47 transmembrane proteins as well. The second most abundant type of HPs (totaling up to 115) was predicted to have enzymatic functions. The remaining numbers of HPs were predicted to be regulatory proteins (52), metal-binding proteins (43) and antibiotic-related (12) (Table 1).

**Table 1:** Number of hypothetical proteins based on predicted functions

**Predicted functions**	**No. of HPs**	**Percentage %**
Unknown protein functions	583	48.5
Signal peptide, transmembrane	221	18.3
Enzymes	115	9.5
Signal peptides	65	5.4
Regulatory proteins	52	4.3
Transmembranes	47	3.9
Metal-binding proteins	43	3.6
Lipoproteins	27	2.2
Transporters	20	1.7
Antibiotic-related proteins	12	1.0
Spore proteins	9	0.7
Others	8	0.7
Total	1202	100.0

### Bleg1_2507 and Bleg1_2337 (putative COX II) Sequence Analyses

Within the pool of metal-binding proteins, HP Bleg1_2507 which consists of 198 amino acids was discovered to possess a copper chaperone Sco1 domain related to Trx-like superfamily from both INTERPROSCAN and NCBI-CDD analyses (data not shown).

Sequence similarity analysis of the HP using BLASTP indicated that Bleg1_2507 is similar to HPs of other *Bacillus* species with 40-50% identities (Table 2). Additionally in the top 10 hits of the BLASTP analysis, results also indicated that Bleg1_2507 shared similarity to electron transport protein Sco1 of various microorganisms. Sco1/SenC protein from *Planococcus antarcticus* DSM 14505 has the highest identity of 47% while the model Sco1 protein from *B. subtilis* (BsSco) surprisingly shared only 35% identity with Bleg1_2507 (Table 2).

**Table 2:** Top 10 hits of BLASTP similarity search against NR database for Bleg1_2507

**Accession No.**	**Description**	**Identity %**	**E-value**
NP 244201.1	Hypothetical Protein BH 3335 *(Bacillus halodurans* C-125)	50	2e-63
YP 176895.1	Hypothetical Protein ABC 3401 (*Bacillus clausii* KSM-K16)	47	1e-43
YP 003425399.1	Unnamed protein product (*Bacillus pseudofirmus* OF4)	45	3e-61
YP 173730.1	Hypothetical Protein ABC 0226 (*Bacillus clausii* KSM-K16)	46	3e-46
NP 243770.1	Hypothetical Protein BH 2904 (*Bacillus halodurans* C-125)	40	9e-46
EIM 07312.1	Electron transport protein SCO1/SenC (*Planococcus antarcticus* DSM 14505)	47	1e-42
ZP 08680789.1	Sco1 family electron transport protein (*Sporosarcina newyorkensis* 2681)	42	8e-44
EIJ 83190.1	Electron transport protein SCO1/SenC (*Bacillus methanolicus* MGA3)	38	2e-35
ZP 10043231.1	Sco1/SenC (*Bacillus* sp.5B6)	35	2e-36
YP 003920672.1	Assembly factor BSco of the CuA site of cytochrome c oxidase (*Bacillus amyloliquefaciens* DSM7)	35	4e-38

**Figure 1:**
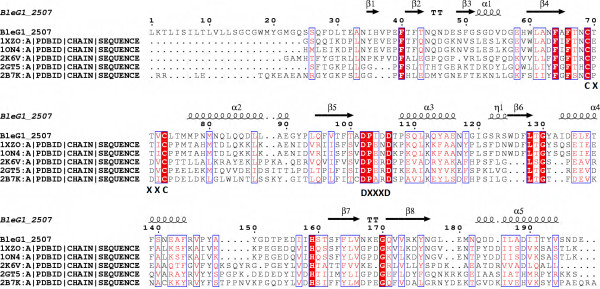
**Multiple sequence alignment of Bleg1_2507 with prokaryotic and eukaryotic Sco templates.** The red boxes indicate conserved amino acids residues. The secondary structures of Bleg1_2337 are shown at the top of the alignment.

Further analysis on Bleg1_2507 using ScanProsite revealed that amino acids 31–196 matched a thioredoxin_2 consensus pattern with total score of 10. Apart from this, analysis on the degree of amino acids conservation in Bleg1_2507 using Consurf revealed that Cys-69, Cys-73 and His-159 were highly conserved and similar to the ones in the top 10 hits from the UniRef 90 database of the Consurf program. The hits include the assembly factor of copper II (CuA) site of COX in *B. atrophaeus* 1942, Sco1 protein homolog from *B. subtilis* (BsSco) and putative uncharacterized protein from *B. clausii* KSM-16. In addition to this, these residues were also identified as potential metal-binding ligands via MetalDetector v2.0 analysis. These metal-binding residues resemble those of Sco1 proteins which similarly contain two Cys and a His residues at their metal-binding sites [33]. Hence, there is a possibility that the conserved Cys-69, Cys-73 and His-159 residues of Bleg1_2507 form the metal binding site of Bleg1_2507. Further analysis on the sequence of Bleg1_2507 revealed that the protein also possessed CXXXC and DXXXD motifs, which are very well preserved in all eukaryotic and prokaryotic Sco proteins [34,35]. Both of the conserved Cys in the CXXXC motif of Bleg1_2507 are made up of Cys-69 and Cys-73 respectively while the conserved Asp in the DXXXD motif are made up of Asp-102 and Asp-106 respectively (Figure 1). The conserved Asp residues of the motif have been implicated in copper ion binding during CuA assembly [35].

**Figure 2:**
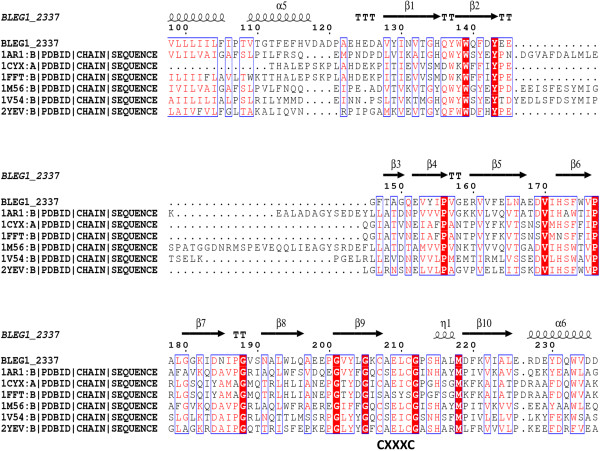
**Multiple sequence alignment of Bleg1_2337.** Yellow boxes indicate the conserved amino acid residues, while red boxes indicate metal-binding residues.

Since the results above highlight the possibility of HP Bleg1_2507 to be a Sco1 protein, a genome search for the sequence encoding the well-documented redox partner of Sco1, COXII [35] was performed. According to [35], 82% of regular prokaryotic genomes possessed the same number of *sco* and *coxII* genes or the absence of both. Based on BLASTP result of Bleg1_2507, there were 5 HPs which exhibited 40-50% of sequence similarity to Bleg1_2507, originating from *B. halodurans*, *B. clausii* KSM-K16 and *B. pseudofirmus* OF4 (Table 2). Search for COXII from the genomes of these microorganisms revealed that COXII was present in these microorganisms with the accession number of NP_243481.1, YP_175889.1 and YP_003425144.1 respectively. This suggested a correlation between these genes and highlights the possible presence of the *coxII* gene in the genome of *B. lehensis* G1. The genome search led to the retrieval of Bleg1_2337 sequence whereby BLASTP analysis on this 344 amino acids protein showed 80% identity to COXII of *B. clausii* KSM-16. ScanProsite analysis showed the presence of Cox2_CuA (PS 50857) domain in Bleg1_2337 spanning from amino acid number 125–236. Three conserved amino acid residues namely Cys-261, Cys-264 and His-265 were identified to be metal-binding ligands. In addition to these residues, Metal Detector v2.0 program predicted Cys-207, Cys-211 and His-215 to be metal-binding ligands as well. ClustalW alignment of Bleg1_2337 showed that its predicted metal-binding residues, Cys-207, Cys-211 and His-215 are very well aligned with those of COXII sequences from other organisms such as *Thermus thermophiles*, *Bos taurus* and *Paracoccus denitrificans* (Figure 2). This result strengthens the prediction that Bleg1_2337 is indeed a COXII protein and that the sets of conserved residues are indeed metal-binding ligands, similar with reported metal-binding ligands of other COXII proteins [36].

Further probing on Bleg1_2337 using Consurf indicated that several hydrophobic residues such as Val-170, Ser-173, Phe-174, Trp-175 and Pro-177 were found to be highly conserved as well (Figure 2). Such hydrophobic residues have been highlighted to play an important role in the hydrophobic interaction with Sco protein [37].

### Homology modeling of Bleg1_2507 and Bleg1_2337

**Figure 3:**
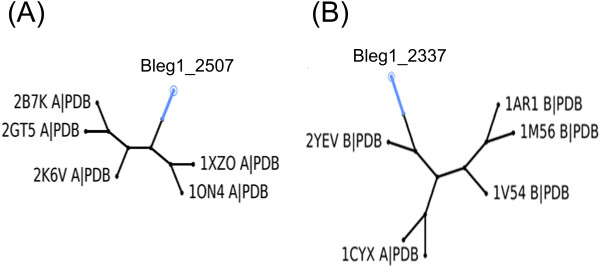
Phylogenetic analysis of (A) Bleg1_2507 and (B) Bleg1_2337 with their possible structural templates.

Potential templates for homology modeling of both Bleg1_2507 and Bleg1_2337 were retrieved using PSI-BLAST [16] search against PDB. Four templates were obtained for HP Bleg1_2507. Alignment of these sequences with Bleg1_2507 showed that Cys-69, Cys-73 of the CXXXC motif, His-159, Asp-102 and Asp-106 of the DXXXD motif of the HP aligned perfectly with all of the potential templates (Figure 1). 1XZO, chain A, which is a crystal structure of a disulfide switch in *B. subtilis* Sco (BsSco) and a well-studied member of the Sco family of COX assembly proteins [37] was selected as the template for homology modeling as it possessed the highest sequence similarity to Bleg1_2507 with 39% identity and lower e-value of 5e-31 compared to other templates (data not shown). In addition to this, parsimony phylogenetic analysis from PHYLIP also revealed that 1XZO is closely related to Bleg1_2507 (Figure 3(A)), lending further credence for it to be used as the template for Bleg1_2507 model generation.

As for Bleg1_2337, six templates were obtained and alignment of their sequences with Bleg1_2337 showed that highly the conserved residues, Cys-207, 211 and His-215 as well as the hydrophobic residues mentioned above were very well aligned. 2yev chain B, which is a crystal structure of caa3-type cytochrome oxidase *Thermus thermophilus* HB8 [36] was selected as the template for homology modeling of Bleg1_2337 as it has the highest sequence identity of 37% and low e-value of 4e-50 when compared to other templates. Phylogenetic analysis from PHYLIP also revealed that 2YEV chain B is closely related to Bleg1_2337 (Figure 3(B)).

**Figure 4:**
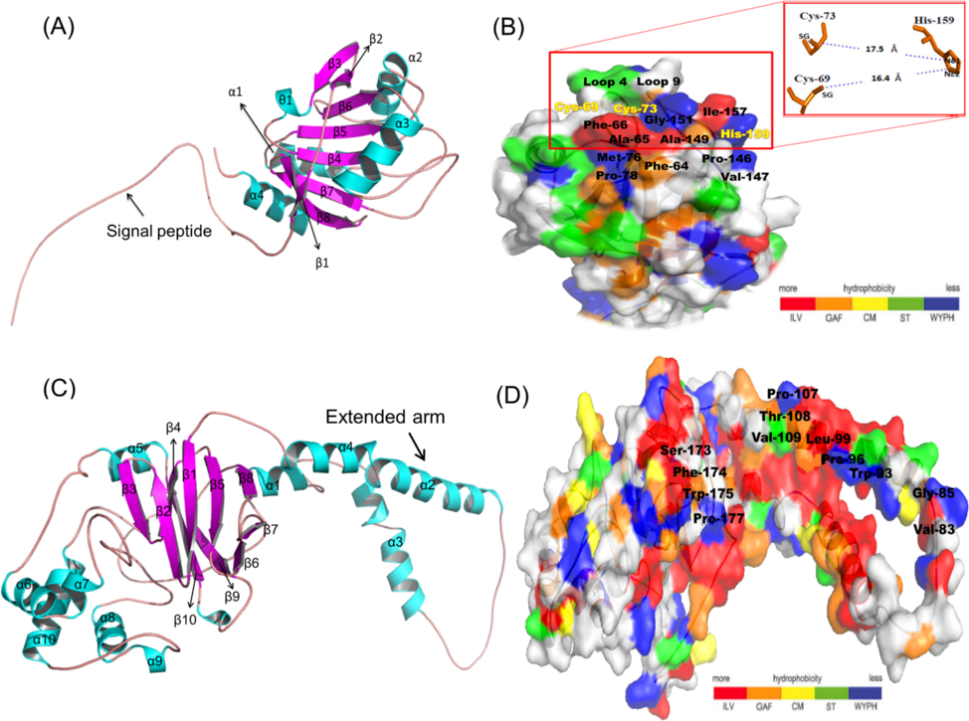
**Ribbon presentation of predicted structures of Bleg1_2507 with extended N-terminus and Bleg1_2337. (A)** Generated model of Bleg1_2507 exhibiting Trx-like global topology, with the presence of four α-helices (cyan) and eight β-sheets (magenta) **(B)** The distances between S of Cys-69 and Cys-73 with Nϵ2 and Nδ1 of His-159 were ~16.4 and ~17.5 Å respectively, which are considered far for metallation process in the predicted Sco protein Bleg1_2507 with the presence of hydrophobic residues on the loop 4 and loop 9 (color-coded and numerically weight hydrophobicity based on [26]) **(C)** Generated model of Bleg1_2337 with a β-sheets cluster at the core of the protein surrounded by 10 α-helices. α1, α2, α3 and α4 form an extended arm which allowed interaction with Bleg1_2507, the predicted Sco protein **(D)** Hydrophobic residues (color-coded and numerically weight hydrophobicity based on [26]) of Bleg1_2337 that might be involved in the interaction with predicted Sco (Bleg1_2507) during metallation process and electron transfer.

The best built models for Bleg1_2507 and Bleg1_2337 (Figure 4(A) & (C)) have the lowest DOPE values and GA 341 scores of one, suggesting that the developed models resemble the native structure and hence reliable. Further supporting results from the superimposition of all Cα atoms of both developed models and templates gave forth low RMSD of less than 0.5 Å, suggesting that the built models are indeed reliable.

### Bleg1_2507 and Bleg1_2337 model refinement and validation

Refinement of the built models using FoldX in YASARA resulted in minimization of the free energy of Bleg1_2507 from 296.2 kcal/mol to 62.2 kcal/mol and from 657.5 kcal/mol to 247.1 kcal/mol for Bleg1_2337.

Evaluations on the quality of the refined protein structures using ERRAT [29] indicated that 84.2% of the amino acids of the refined Bleg1_2507 model were located in the acceptable region within 95% confidence limit, as opposed to only 70.5% before refinement. As for refined Bleg1_2337 model, 85.4% of its amino acids were located in the acceptable region within the 95% confidence limit, as opposed to only 75.4% before refinement. These indicated that the refined protein models for Bleg1_2507 and Bleg1_2337 (Figure 4(A) & (C)) were precise and reliable since the frequencies of atom randomizations were low. Further supporting results from PROCHECK Ramachandran analysis on refined Bleg1_2507 model revealed that 85.7% of the residues were located in favored region, 12.3% in additional allowed region, 1.3% and 0.6% in generously allowed and disallowed regions respectively. As for Bleg1_2337 refined model, 87.7% of the residues were located in favored region, 10.3% in additional allowed region and 2% were located in generously allowed region. Overall, both ERRAT and PROCHECK evaluations further reaffirmed that both of the predicted protein models were reliable.

### Probing the structures of Bleg1_2507 and Bleg1_2337

Overall, the built model for HP Bleg1_2507 showed that it adopted a global topology unique to Thioredoxin (Trx)-like superfamily of proteins including Sco proteins whereby four β-sheets (β4, β5, β7 and β8) are flanked by three α-helices (α1, α3 and α4) at the core of the structure (Figure 4(A)). This is similar to Trx-like superfamily members which have a characteristic, common core βαβαββ secondary structural pattern with different insertions of secondary structural elements to distinguish the various structural families such as thioltransferases, glutaredoxins, bacterial arsenate reductases and disulfide bond isomerases. They are in general involved in cellular thiol-redox reactions to maintain the reduction and oxidation of proteins or certain small molecules [38]. Other than this core structure, Bleg1_2507 also consisted three flanking β-sheets (β1, β2 and β3), a 3_10_ –helix at the N-terminus and a hairpin formed by β2 and β3, which are the additional secondary structure elements only present in Sco proteins [39].

Other similarities between include the presence of a transmembrane region in the N-terminal of Bleg1_2507 in which an external loop comprising of 25 amino acids at the N-terminus could be observed (Figure 4(A)). SigCleave analysis from EMBOSS [21] revealed that the amino acids from Leu-4 to Cys-16 in this loop were predicted to be a signal peptide, which can be subjected to cleavage to give forth the mature protein. Previous study has shown that in the Sco protein of *B. subtilis*, (BsSco) the first 20 amino acids are located in the transmembrane and Cys-16 is the signal peptidase II recognition site. To this cleaved site a diacyl glycerol moiety will be attached to the protein and the post-translationally modified protein will subsequently be able to anchor to the membrane through the attached lipid by covalent bond [39]. This in turn highlights that Cys-16 in HP Bleg1_2507 is most probably a cleavage recognition site similar to the one found in BsSco for processing of the native Sco protein before it can be partially integrated into the membrane.

Unique to Bleg1_2507 however, there is an α-helix (α2) near loop 4 and a β-strand (β6) that are parallel with β5 and β4 (Figure 4(A)), which are not present in Trx. In addition to this, the location of the metal-binding ligands in Bleg1_2507 is different from the solution structure of BsSco*.* For instance, in the predicted model of Bleg1_2507, both metal-binding Cys-69 and Cys-73 residues were located in loop 4 (situated in between α1 and β4) while His-159 was positioned in loop 9 (situated in between α3 and β7) (Figure 4(B)). In BsSco on the other hand, its metal-binding residues, namely Cys-45 and Cys-49, were located in loop 3 and the conserved His-135 was located in loop 8 [6].

Looking closely at the predicted metal-binding cavity of Bleg1_2507, it featured the highly conserved Cys-69, Cys-73 (of the CXXXC motif) and His-159 residues. These residues which are strictly preserved in all Sco proteins have been implicated to be responsible in copper-binding and redox reaction [39]. In proximity to the CXXXC motif, a hydrophobic groove which is formed by Phe-64, 66, Ala-65, Val-72, Met-76, 77 and Pro-78 was observed (Figure 4(B)). Besides this, another hydrophobic groove formed by the His-ligand loop consisting of Val-146, Pro-147, 154, Ala-149, Gly-151, Ile-157, 158, and Phe-163 was also observed (Figure 4(B)). Both of the hydrophobic grooves formed a hydrophobic pocket which may allow proteins and small molecules such as copper ion to be accommodated [6].

According to Balatri *et al*., 2003 [6], the interaction of hydrophobic residues within the protein are essential in the metallated state of Sco, because it is able to stabilize the metal-ligand region of the protein, which are located far apart from each other within the two hydrophobic grooves, by coordinating the metal-ligand geometry. Based on the Sco1 protein crystal structures from *B. subtilis*[6], yeast [10] and human [40] the distances between the N atoms of His residues and the S atom of Cysteine pair are in the range of 10–19 Å. In the case of Bleg1_2507, the distance between Nϵ2 of His-159 and S of Cys-69 was ~16.4 Å, while the distance between Nδ1 of His-159 and S of Cys-73 was ~17.5 Å (Figure 4(B)), similar to the range noted above for the respective Sco1 proteins from *B. subtilis*, yeast and human. These distances are considered far for metal-ligand interaction in the apo-form state of the predicted Sco protein. It has been proposed by Balatri *et al*., 2003 [6] that the hydrophobic residues in the hydrophobic grooves of Bleg1_2507 might be responsible in regulating the appropriate geometric coordination for metal-ligand interaction to occur. According to Andruzzi et al. [41], the oxidized BsSco was coordinated to Cu(II) by a distorted tetragonal square planar, in which the Cys-45, Cys-49 and His-135 are ligated equatorially and water was ligated axially to the Cu(II) respectively. Hence, Cys-69, Cys-73 and His-159 of the predicted Sco of Bleg1_2507 was proposed to coordinate the copper through square planar geometry. Based on these observations, we propose that this particular HP is potentially a Sco protein with an ability to bind and chaperone copper to the CuA site in COXII.

### Interaction between Bleg1_2507 and putative COXII redox protein, Bleg1_2337 and their possible mechanism of action

**Figure 5:**
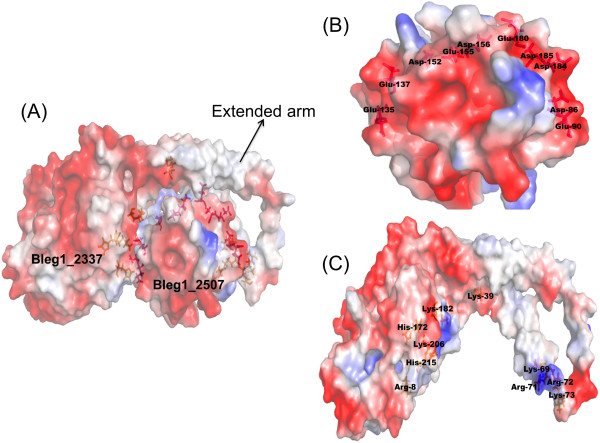
**Docking between the generated models of Bleg1_2507 and Bleg1_2337. (A)** Interaction of predicted models of Bleg1_2507 and Bleg1_2337 with the assistance of acidic residues of Bleg1_2507 **(B)** and basic residues of Bleg1_2337 **(C)** in addition to the hydrophobic residues.

To test the above hypothesis, HP Bleg1_2507 was docked to its predicted redox partner, Bleg1_2337, a putative COXII protein to investigate possible interaction between these two proteins. Docking results showed that Bleg1_2507 was indeed able to interact with Bleg1_2337 with lowest energy of −1163.8 KJ/mol. From the results, Bleg1_2337 could be seen embracing Bleg1_2507 with an extended arm (Figure 5(A)). Refinement of the docked protein complex successfully decreased the potential energy from −257189.2 KJ/mol to −295640.7 KJ/mol signifying favorable protein conformation.

Inspecting the interface of both interacting proteins, Bleg1_2507 possessed a set of acidic residues such as Asp-47, 55, 56 and 126, Glu-48, 141 and 169 on its surface (Figure 5(B)). This observation lends support to ProtParam analysis which revealed that Bleg1_2507 has an acidic pI of 3.95, indicating this HP has abundant number of acidic residues. Its putative COX partner, Bleg1_2337, on the other hand, possessed a set of positively charged, basic residues on its surface such as Lys-3, 34, 69,73, 182 and 206, Arg-8, 71 and 72 and His-215 (Figure 5(C)), which gave the protein a basic pI value of 4.21 according to ProtParam analysis. The presence of these oppositely charged residues on the respective proteins undoubtedly facilitated ionic interaction between the two macromolecules. Such ionic interaction between Sco and COX proteins has been similarly observed and was proposed to generate a charged-mediated Sco-apo-CuA located in the COX protein which will allow copper exchange to occur [39].

Based on the mechanism proposed for BsSco protein, for copper to be bound and later transferred from Sco to COX, both of the conserved Cysteine residues in the CXXXC motif of BsSco will be firstly reduced to di-thiol state via formation of SH-SH bond. This will result in a reduced BsSco protein that is able to strongly interact with Cu(II) ion to form BsSco-Cu(II) complex. From the BsSco-Cu(II) complex, the metal ion will then be transferred to its intended target i.e. apo-subunit II of COX via association of both of the proteins (BsSco and COXII). In this process, the Histidine residue was postulated to play an important role in this second phase of copper coordination by regulating the structural dynamism of bacterial Sco [6]. During association of both of the proteins, apart from metal ion transfer, an oxidation-reduction (redox) process also takes place via electron transfer [39]. During interaction of the proteins, BsSco will undergo oxidation by releasing electrons which will be subsequently delivered to the binuclear CuA site of COXII. Consequently, the received electrons in the CuA site of COXII which made up of Cys-217, 221 and His-215 will reduce Fe(III) to Fe(II) or Cu(II) to Cu(I) [39]. The di-thiol state of BsSco is vital in this electron transfer process and maintaining the redox state of the partner.

In the case of Bleg1_2507, it is therefore highly possible that it may possess redox properties by potentially acting as a thiol disulfide oxidoreductase. Similar to BsSco, both the conserved Cys-69 and Cys-73 in Bleg1_2507 may also form the SH-SH bond and give forth the di-thiol state of the protein. From this point onwards, Bleg1_2507 HP may be able to undergo oxidation to release electrons which are subsequently delivered to the binuclear CuA site of reduced COXII of Bleg1_2337 which were made up of Cys-207, 211 and His-215 and Cys-261, 264 and His-265 (highly similar to the other documented COXII proteins), to reduce the respective metal ion at the site. This postulation is further supported by the presence of hydrophobic grooves in HP Bleg1_2507 which form an extensive uncharged surface surrounding the active disulfide bond formed by Cys-69, Cys-73 and His-159 encompassed in a solvent exposed environment which might facilitate the electron transfer process, similar to the features observed in BsSco [35].

It is worthy to note that other than binding metal ions, the conserved Cys pair and His-ligand loops of Sco proteins are important for Sco to interact with other proteins as well [33]. This feature was clearly observed in our docking results where these residues of HP Bleg1_2507 are shown to interact with putative COXII (Bleg1_2337) via hydrophobic interaction, stabilized by uncharged surface features surrounding the hydrophobic pocket between loop 4 and loop 9 (Figure 4(B)). In the Cysteine pair loop (loop 4), it possessed uncharged polar residues such as Thr-67, 70, 71 and 75 and Asn-68. While, Thr-153, 161 and Ser-160 were located in the His-ligand loop (loop 9). Similarly in its redox partner, the putative COXII (Bleg1_2337), crucial hydrophobic residues such as Val-82 and 109, Gly-84, Trp-92 and 175, Ile-95, Pro-96 and 107, Leu-99, Phe-106 and 174, Thr-108, Ser-173 and Pro177 may have contributed to the interaction of both proteins (Figure 4(D)), in accordance to other similarly reported study [39]. All of these residues are crucial in maintaining the intactness of Sco-CuA protein complex for electron transfer [39].

### Structural and functional comparison of Sco proteins

**Figure 6:**
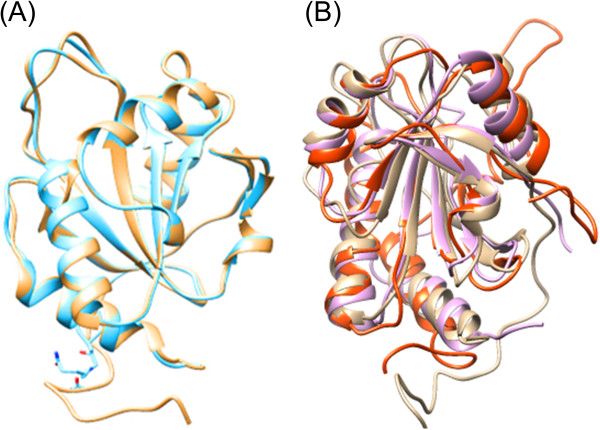
**Structural alignment of Bleg1_2507 protein model with BsSco, human and yeast Sco1 proteins. (A)** Superimposition of Cα atoms between Bleg1_2507 (brown) and BsSco [1XZO, Chain A] (blue), **(B)** human Sco1 [2GT5, Chain A] (orange) and yeast Sco1 [2BTK, Chain A] (magenta) proteins exhibited structural resemblance across biological taxa.

Comparing the sequences of the predicted Sco Bleg1_2507, BsSco (1XZO, chain A), human Sco1 (2GT5, chain A) and yeast Sco1 (2BTK, chain A), these proteins share many conserved residues regardless of their taxonomic and sequence differences (Figure 1). Superimposition of predicted protein model of Bleg1_2507 with the 3D structure of BsSco showed that they are highly similar with RMSD value of 0.286 Å (Figure 6(A)). Interestingly, superimposition of predicted protein model of Bleg1_2507 with the 3D structures of human and yeast Sco1 showed that their structures are comparatively similar as well even at RMSD values of 1.215 Å and 1.060 Å respectively (Figure 6(B)).

It is important to note that Bleg1_2507, BsSco and Sco1 of yeast contain the same number of β-sheets and α-helices i.e. eight and four respectively. The 3D structure of human Sco1 (2GT5, chain A), however, contained only six β-sheets. The two additional β sheets in Bleg1_2507 are β2 and β3, which forms the hairpin structure located before the 3_10_- helix at the N-terminus.

In addition, although Bleg1_2507 shares relatively low sequence identity to BsSco (47%), human Sco1 (2GT5, chain A) (27%) and yeast Sco1 (27%), the predicted metal-binding residues of Bleg1_2507 i.e. Cys-69, Cys-73 and His-159, were located at flexible active loops (loop 4 and 9) similar to other Sco proteins across biological taxa.

It is indeed interesting to note that despite their remarkable structural similarity, Sco proteins are rather diverse in function. In eukaryotes, Sco proteins are involved in the assembly of the CuA cofactor of mitochondrial cytochrome c oxidase, redox signaling and regulation of copper homeostasis [13]. In prokaryotes, Sco proteins are more promiscuous in functions. While some are required for COX biosynthesis, others have been implicated in different processes such as copper delivery to other enzymes and protection against oxidative stress [33]. More investigations as to the physiological function and mechanism of Sco proteins will be useful in determining its exact role and importance in the system biology of an organism.

## Conclusions

In this study, genome mining of hypothetical protein sequences of *B. lehensis* G1 alkaliphile led to the discovery of Bleg1_2507 which showed the presence of a Trx-like domain linked to Sco protein. Showing only 35% of similarity to BsSco, the prokaryotic model of Sco, the 3-D model of Bleg1_2507 was built by homology modeling. The built protein model showed good preservation of the βαβαββ core structure (characteristic of many Trx-like redox proteins), three flanking β-sheets, a 3_10_ –helix at the N-terminus and a hairpin (characteristic of Sco proteins). Unique to Bleg1_2507, there is an α-helix (α2) near loop 4 and a β-strand (β6) that are parallel with β5 and β4, which are not present in Trx. Another distinct feature of Bleg1_2507 compared to BsSco is the difference in the location of the metal-binding ligands in Bleg1_2507. Docking simulations interestingly provided a view of Bleg1_2507 in association with its putative COXII redox partner, Bleg1_2337, where the latter can be seen to hold its partner in an embrace, facilitated via hydrophobic and ionic interaction between the two proteins. Although Bleg1_2507 shares relatively low sequence homology to BsSco, interestingly, it is structurally similar to the protein as well as other Sco proteins across biological taxa. This observation highlights that despite its varying functions in prokaryotes and eukaryotes, Sco proteins are structurally well preserved.

Based on all the results obtained, we hereby propose that HP Bleg1_2507 of *B. lehensis* G1 is a Sco protein which is able to interact with COXII, its redox partner. Hence, it is highly possible that HP Bleg1_2507 may possess metallochaperone and disulfide redox functions similar to other documented bacterial Sco proteins. It is hoped that the predicted structure and function of Bleg1_2507 from the hypothetical protein dataset of *B. lehensis* G1 will help to spur the search for other physiologically relevant proteins among the so-called “orphan” proteins of any given organism as well as direct future biological studies in improving our understanding of COX complex assembly process.

## Competing interests

The authors declare that they have no competing interests.

## Authors’ contributions

TSH carried out bioinformatics analyses and clustering of all the hypothetical proteins, homology modelling and protein docking in this study. TSH and YMN drafted the manuscript. ABS, MBAR, RAK, ALTC, AMAM, NMM revised and proofread the manuscript. YMN conceived the study and participated in its design and coordination together with ABS, MBAR. RAK, ALTC, AMAM gave technical advice to the study. All authors read and approved the final manuscript.
